# Changes in computed tomography and ventilation/perfusion mismatch with positive end-expiratory pressure

**DOI:** 10.1186/cc13466

**Published:** 2014-03-17

**Authors:** DS Karbing, M Panigada, N Bottino, E Spinelli, A Protti, SE Rees', L Gattinoni

**Affiliations:** 1 Aalborg University, Aalborg, Denmark; 2 Fondazione IRCCS Ca Granda Ospedale Maggiore Policlinico, Milan, Italy

## Introduction

The purpose was to compare effects of PEEP on computed tomography (CT) and estimated ventilation/perfusion (V/Q) mismatch. Previously, an oxygenation-based method was shown more related to the CT-measured effect of PEEP than lung mechanics [1], indicating lung aeration is better quantified using V/Q mismatch. Pulmonary shunt and low and high V/Q mismatch can be estimated from varying FIO_2 _and measuring ventilation and blood gas contents [2].

## Methods

Preliminary results in six ARDS patients. CT scans were taken in static conditions at PEEP 5, 45 and 15 to 20 cmH_2_O. V/Q was estimated at 5 and 15 to 20 cmH_2_O as: shunt, low V/Q as alveolar to lung capillary PO_2 _difference (ΔAcPO_2_), high V/Q as alveolar to lung capillary PCO_2 _difference (ΔAcPCO_2_) [2]. Nonaeration, poor aeration, and normal aeration plus hyperinflation were calculated from Hounsfield units. Aeration and V/Q were compared (Pearson, ρ).

## Results

**Figure 1:**
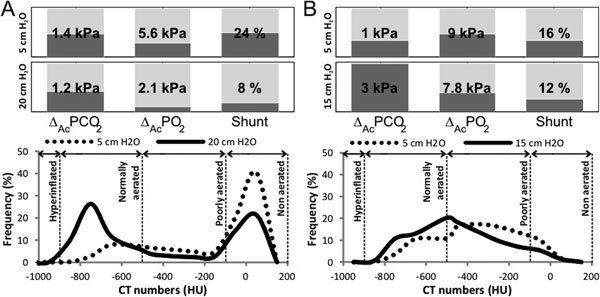
**Gas exchange and CT for patient improving (A) or worsening (B) with PEEP change**.

PEEP improved V/Q in four patients, shunt reducing 7 to 42% with no/small increase in ΔAcPCO_2_. Two deteriorated, with large ΔAcPCO_2 _or shunt increase. No systematic changes in ΔAcPO_2 _were seen. Figure 1 shows response to PEEP in two patients. Changes in nonaerated regions and shunt were correlated (Δ = 0.94, *P *= 0.002). No correlations were found between poorly aerated regions and ΔAcPO_2 _(Δ = -0.09, *P *= 0.84) or hyperinflated regions and ΔAcPCO_2 _(Δ = 0.07, *P *= 0.88).

## Conclusion

In these preliminary cases, changes in shunt and nonaerated tissue correlated well. However, results indicate poor agreement between changes in low and high V/Q and lung morphology.
